# Experiences of a Teacher in Relation to the Student’s Feelings of Learned Helplessness

**DOI:** 10.3390/ijerph17218280

**Published:** 2020-11-09

**Authors:** Gustavo González-Calvo

**Affiliations:** Department of Musical, Plastic and Body Expression, University of Valladolid, 34004 Palencia, Spain; gustavo.gonzalez@uva.es

**Keywords:** permanent education, motor incompetence learned, democratic learning, autoethnography

## Abstract

This paper is based on the concern of a novice physical education teacher to reinforce the self-esteem and motor competence sensations of students during lessons. This concern arises from the experiences gained as a student. I draw on autobiographical narratives to delve into how these experiences led me to develop a feeling of learned incompetence, a sense of failure within the educational system and, consequently, an obvious difficulty to shape my personal and professional identity. However, it is these same experiences that condition professional development and teaching practice. Thus, I attempt to break from pedagogical models and to offer a dignified and democratic education to students. I attempt to engage the reader by communicating the subjectivity of different moments in a provocative, fragmented, physical, and emotional manner. Thus, I share the concerns, reflections, and manner of working, as a teacher, in the form of autobiographical reports and class journals. The intention is to understand how this manner of working responds to the feelings of incompetence learned by school children.

*“Among all the reasons, fear is the only one that is wrong”*

(Gustavo González-Calvo)

## 1. Introduction

In-service teacher training is an excellent means of professional development, understood as an attitude of constant learning. From this perspective, training and professional development form a necessary partnership for the teaching profession. 

One way of meeting this requirement is to begin with the teacher’s reflective practice. This allows the teacher to carry out their professional self-training to embark on a path of searching for themselves and reasons for their actions. This also provides teachers with the capacity for problem solving and to face up to uncertainties experienced in the classroom on a daily basis and, in short, transforming and improving their practice.

Following a reflective model also allows one to go beyond the perspective of the competent technician to implement a reflective practice, and break with the simple and trivial vision that often accompanies the teaching profession. Reflection emerges as an essential tool for the personal growth and professional development of the educator. It is important that teaching, and therefore education, cannot and must not be reduced to the mere application of instructions given from outside the school context.

By adopting this stance, the teacher who is reflective establishes themselves as a professional capable of mastering their own evolution, building new skills and knowledge to extend those they have previously acquired, in addition to their own experience. Consequently, this article aims to awaken in students who are currently engaged in their initial training, and future physical educators, the reflexive spirit that allows them to question, rethink, and accommodate their pedagogical practice with the intention of providing answers, in the best possible way, to all schoolchildren.

## 2. Learned Helplessness and Physical Education

In the field of education, there is a well-known phenomenon whereby schoolchildren may become aware that they are not very competent in certain activities, which undoubtedly includes those related to the motor field. In this sense we can speak of learned helplessness [1], because some students show a certain predisposition to believe that they do not have control over the situation in which they find themselves. In the field of motor skills, this term has been popularized as learned motor incompetence [2,3], that is, the phenomenon by which being unfit for certain physical activities is assumed to be unavoidable. Thus, a poor disposition towards learning and physical practice can lead the student to develop a negative self-concept that results in low self-esteem [4], originating a vicious circle that is very common in our classes. Moreover, it is also clear that it does not favor the involvement and participation of the student in the teaching/learning process. 

When the experiences are negative—that is, when schoolchildren are humiliated, compared, frightened, and restrained—the self-esteem developed in the child is based on submission, passivity, and disinterest. As a result, these students become “physical education objectors” [5], p. 34. These objectors, on many occasions, are no less capable than other schoolchildren. However, because of the treatment they receive from their teachers as a result of the dominant motor culture that is reflected in the subject of physical education (henceforth, PE), or any other reason, they develop this feeling of learned helplessness. 

The pertinent characteristics of PE in Spanish schools include preprimary, primary, and secondary stages. Different strands in PE are physical fitness, sports, and corporal expression. Three hours of PE per week are taught in primary schools, and 2 h per week are taught in secondary schools. A mix of teaching and coaching orientation is embedded within the different contexts. Teachers, via their approach to learning activities, can affect the attitude of students. Thus, when the physical exercise is deemed to be satisfactory by the practitioner, a favorable judgement of the skill itself and a positive attitude towards it is produced. However, the attitude towards practice becomes unfavorable when the conditions of the task environment exceed one’s possibilities of execution, thus producing a lack of self-confidence [6] and personal dissatisfaction [7]. Therefore, it is important that the teacher approaches tasks in such a way that students can experience successful situations and learn to enjoy being competent, regardless of their age, level, interests, abilities, and previous experiences. According to Ruiz Pérez [3], students who continually fail tend to be considered as lacking competence, and show levels of stress and anxiety that are not favorable to learning. Pennac warns of this risk [8], and explains that “some children are quickly persuaded that this is the way things are, and if they do not find anyone to disappoint them, as they cannot live without passion, they develop, for lack of something better, the passion of failure” (p. 53). 

In the area of PE, in particular, teachers’ previous knowledge can affect their approach to their classes and, as a result, the predisposition of their students towards physical activity. Most teachers in the field of PE have had rewarding and positive experiences as schoolchildren in the area of movement and bodywork [9]. This situation means that they can be considered to be winners of the educational system [10]. 

Within this search for the best conditions and optimum learning environment, it is therefore particularly important to pay attention to the way in which students face the activities proposed by the teacher, the difficulties they encounter, and the attempt to offer them the most appropriate responses to achieve the objectives set. This situation could mistakenly lead to the view that it is important not to leave room for error within the teaching/learning process. However, errors (regardless of whether they are inevitable or necessary) will always be present in the process itself; thus, the ideal is to teach students to assume errors are inherent and then control them. It is therefore a question of ensuring that students are able to better understand the nature of their actions, learning from them and from the corrections they make. The intention is to create and encourage a space free of threats [11] so that schoolchildren can have the opportunity to experiment within an environment that fosters confidence and self-esteem. 

This is even more important in a subject such as PE, due to its public and easily observable character [12], in which the schoolchild has to show himself in front of a group of peers. This is a situation in which less competent students tend to feel ashamed or afraid of showing themselves in public, knowing that others will form an opinion and expectations about these attitudes, in addition to about the person himself [6].

Although several articles have been published about learned helplessness and PE, the majority of these are quantitative. As far as the author of the current study is aware, this is the first article about this topic using a qualitative methodology. 

The main objective of this article is to determine, through their class diaries and life story, how new PE teachers build and focus their teaching practice based on how they lived as a student. More specifically, we set ourselves the following objectives: (a) To understand the personal and pedagogical reasons for the teacher’s practice to address the incompetence learned among students; and (b) to reflect the manner in which practice is (re)constructed, and teaching and education are understood, in the first years of the teaching profession with regard to the issue of learned incompetence. 

## 3. Materials and Methods 

### 3.1. Participant 

At the time of this research, I was a novice PE teacher who developed the profession in primary education, secondary education, and university. I began studying a university qualification in chemistry; however, for several reasons (e.g., being older than average, influence exerted by some professors, and readings which helped me understand PE as an excellent pedagogical tool not only for academic learning, but also for moral, social and democratic learning [6,13]), in 2003, aged 25, I changed from chemistry to a Degree in Teaching in the field of PE and Sports. It was then that I developed an (auto)-reflective interest and, in an attempt to understand and improve my practice, I started writing autobiographical narratives. 

Having completed my studies in 2006, I took a second Degree in Physical Activity and Sports Sciences (for Secondary Education). Once completed in 2009, I started preparing for my exams in Public Service in Primary Education (filling a vacancy as a supply teacher for the school year 2009/2010) while simultaneously starting my doctoral studies, obtaining my PhD in 2013. In 2010 I entered the competition exams for Public Service in Secondary Education, working as a supply secondary teacher during the year 2010/2011. In 2011, I finally passed the selection process, obtaining a permanent post as a civil servant in primary education, work I have undertaken until now. Moreover, since 2011, my job as a primary education teacher has been combined with my activities as a part-time lecturer at university. This is the main reason why I decided to publish the article in 2020, even though the last data collection was conducted in 2011: because I believe that it can be a useful resource for students who are in their initial training and future physical educators.

### 3.2. Data Collection

This investigation adopts a phenomenological, narrative, reflexive, and autobiographical perspective to the personal and pedagogical life of the participant [14,15,16]. This perspective provides the foreground for the subject of study, thus making his voice heard as an excellent tool for research and interpretation. Therefore, through a deeply reflexive and introspective process, the teacher can face his own experiences, extracting meaning from them in an active way. 

The data used in this study was derived from two phenomenological sources: an autobiographical diary, which captured the main reasons that led me to become a PE teacher, and a field diary, in which I collected experiences and reflections regarding the objective of his research. 

The narrative of my own life, both personal and professional, began during my initial training and first approach to school, which contributed to the configuration of its current subjectivity (“subjective warrant”) as a teacher [17,18]. These beginnings awakened a process of prejudice, fear, feelings, values, choices, and expectations which were gradually added to the autobiographical account. As a praxis, from this critical, conscious (re)construction of my bio-pedagogical journey, from present to past, I extracted information about my professional identity, in an attempt to understand how it had developed in the different social and educational context in which he took part [19,20]. In addition, my life narrative allowed me to voice my own consciousness [21] thus strengthening the narrative’s credibility [22,23,24].

The other element used for data collection was the class diary. Several authors [9,25,26,27] believe that diaries are useful tools, stimulating critical and reflective processes, because they help to broaden the perspective with regard to events happening in the class, and help teachers to deepen the analysis of their own actions and ideas about teaching–learning processes.

The first experience with the class diary of the researcher-participant (R-P) appeared, as stated above, during the initial stage of training as a PE teacher, in 2005. This allowed me to test its usefulness as a tool for formation and investigation, because it enabled me to gather significant data, reflect upon it and, at the same time, to analyze and systematize it. The R-P then continued to use this instrument to collect information, having completed several diaries during a period of five years (a) as a primary teacher during the years 2009–2010, 2011–2012, 2012–2013, and 2013–2014; and (b) as a secondary teacher and a part-time lecturer at a Spanish university during the year 2010–2011. 

All of the participants of the autoethnographic study provided written informed consent. The study was performed in accordance with the Declaration of Helsinki, and the protocol was approved by the Ethics Committee of the University of Valladolid (HRS4R).

### 3.3. Data Analysis

Both the autobiographical narration and the class diaries (primary, secondary and university diaries) were “narratives as practices” [28] which, in themselves, lead to “acts of meaning” [29] about the teacher’s professional identity as a narrator of my “self” and my own experience [30]. Furthermore, they also helped me in my analysis, so that I could establish a triangulation and coherence in the theoretical–practical relationship between both sources of information [31,32,33,34,35], something that also increased the credibility and transferability of the study [22,23,24]. 

The process of analysis first involved subjecting the contents of the diaries and the autobiographical narrative to a double, categorical analysis [35,36,37]. These were then compared and contrasted in an attempt to find similarities related to the formation of the teacher’s identity in the process of establishing relationships with the students. According to Polkinghorne [36], the analysis of content can be carried out: (a) inductively, identifying data that derives from narrative sources; and/or (b) deductively, using data that stems from theories previously established in the relevant literature. These two processes were followed in this study during both stages. 

The triangulation of the sources of analysis showed how diaries and life histories complemented each other by comparing daily perspectives with remembrances from past experience, thus enabling the analysis of the same event from two different temporal perspectives. In addition, data underwent an additional process of auto-remembrance and auto-observation, following Spry’s method [38], who wrote an autoethnography report about “embodied methodological praxis”. Consistent with this narrative, the researcher systematically switched between “being here” and “being there” in an attempt to reveal relevant patterns of a constantly changing social and psychological “self”.

Finally, to avoid prejudice during the analysis, I also turned to experts with whom I collectively selected and classified the data reaching credible, reliable conclusions [39].

## 4. Results and Discussion

The results of the study were grouped into three large blocks: (1) the reflections on learned incompetence from the personal biography; (2) the process followed in the (re)construction of the teacher’s pedagogical practice to address the feelings of learned incompetence of the students; and, (3) opening up perspectives on learned incompetence among future PE teachers.

### 4.1. Reflection on Incompetence Learned from Personal Biography

Looking back, my memories of PE classes can be grouped into two very different categories. Thus, in the first of these, enrolled as a student in a public school, I remember myself as a happy child, going to school with enthusiasm and a desire to learn and play with others. I do not remember that the classes in the area followed any particular system, but rather they were a kind of “prolongation of playtime”. However, it was a space and a time in which we all had the opportunity to feel part of the activities, regardless of issues related to ability, sex, or any other factor.

A few years later, I changed schools, this time to a religious school. It was a predominantly male school, and in my class all of the students were boys. The homogeneity of the sexes was reflected in the way the school approached teaching. We all received the same responses from the teachers. Thus, for me and most of my classmates, the educational help provided by the PE teacher, his possible emotional support, and his pedagogical approach were the same, regardless of who was receiving it. 

However, there was a small group of schoolchildren who were treated differently, sometimes for reasons that had little or nothing to do with the school environment. Thus, the students who stood out most in terms of their motivation did so not because the teacher was able to get the best out of them and, consequently, the remaining students were inferior. Rather, these favored students participated in the school’s sports teams and, because one of the pillars of the school was justified in terms of the competitive level of these teams compared to those of other schools, the students received differentiated treatment. 

Similarly, the classes were reduced to an evaluation in the form of a series of tests and physical examinations in which the only students that were reinforced were precisely those that received the most positive reinforcement from the teacher. Students with low expectations, including myself, tended to confirm the expected results because they had not enjoyed the appropriate conditions to improve their physical performance. In the lessons during which we had to show our level of physical condition by passing certain physical tests, I tried to improve, because it seemed that I had to prove something to my new classmates for whom I did not appear to exist (in contrast to the repeaters, who existed at all times). I had to show myself, in particular, to others: derogatory comments by a small group of colleagues towards the majority led to feelings of shame each time we were publicly graded. Adding to my apparent feeling of low self-worth, which was partly caused by the education I was receiving at the school, was a sense of contempt for my body and feelings of sadness and inferiority. I didn’t like the PE classes, I was afraid of being exposed for my incompetence in some of the required tests, and I didn’t see the point of what was being asked of me.

Thus, and despite the fact that I was a physically active boy (I took part in several extracurricular physical activities, even though these were not part of the dominant motor culture of the school, i.e., football and basketball), my conviction that the teacher expected little from me limited my progress and reinforced the idea that I was not competent for physical exercise. 

In addition, the behavior shown by the few privileged students who enjoyed the teacher’s attention did not reinforce the feeling of esteem and personal worth of the remaining students (e.g., “innocent” aspects, such as dividing groups to make teams, or letting only those who are considered to have a moderate skill level play, can dent one’s self-confidence). This “noisy minority” [36] (p. 182) conditioned the opinion of the remainder of the group, including myself, and led, indirectly, to motivating me to outdo myself in the sessions, to wanting to do better, to trying to be part of that select group. However, not feeling accepted among the chosen ones led to feelings of sadness, inferiority, and even contempt for my own body. It also led me to despise the PE classes, to fear being exposed for my supposed incompetence in some of the required tests, and to not see the meaning of what was being asked of me there. I became, therefore, an objector to PE. And although I was moderately good in PE classes, I gradually developed feelings of motor incompetence.

Therefore, and taking into account that adolescence is a complicated stage of one’s life, full of changes during which the self-esteem of a young person is forged, these negative feelings towards myself shaped a large part of my personal identity in those years. Because I was aware of the problem via first-hand suffering meant that this personal identity subsequently conditioned the development of my professional identity and my teaching practice, an aspect which is dealt with in the following sections (Autobiographical account, 2010).

The configuration of the concern to respond to the feelings of low esteem that schoolchildren often have in PE classes, based on their experiences as students, leads me to want to break with the teaching models received, and encourage a more dignified and democratic practice of teaching. 

Thus, and as a result of this past, I corroborate that the real capacity that an individual possesses depends, fundamentally, on his or her own perception of competence. This perception is directly linked to involvement in physical practice, in which I observed that schoolchildren who have a greater perception of motor skills are those who are most involved in physical exercise [37,38,39], the objection and distance that I felt as a schoolchild towards the subject, and the lack of identification with it can be explained. 

Therefore, the first concern I have as an educator during my initial training stage is focused on reinforcing each schoolchild’s idea of competence, as described in the following extract from my diary as a specialist teacher:

*“Among the student body there are several children who are considered unfit for PE. In some cases, their physical limitations are compounded by the teacher’s attitude, which does not encourage them to develop a positive self-concept that would lead them to be competent and thus to live a better future life with themselves. In the planning and implementation of my sessions I have not only tried to work on improving the relationships between the different members of the group, without giving importance to the partner with whom we have to work, regardless of their psychic, physical, gender, ability or any other characteristics. I have also tried to raise the self-esteem of those who consider themselves less motor capable, making them more involved and more confident of their possibilities”*.

(Teacher’s Diary. March 2005)

From this first approach to school reality, the conviction has emerged that it is important to detect and get to know these “fragile subjects” [40] p. 34, and to be able to teach in accordance with their needs, interests, concerns, and problems [41], because it is important that motor skills are strongly imbued with personality. My intention to detect and respond to fragile students is evident during my term as an interim teacher, in which the trail of the past lived as a student can be appreciated and in which I am more aware of the threat implied by the schoolchild developing a distrust of his own possibilities, as can be seen in the following story:

*“I am very sad to know how quickly children learn bad things and how difficult it will be for them to change their concept of themselves as they grow up. If they don’t experience success, if they don’t have a good relationship with their body, if their self-image is distorted, they will have a hard time dealing with it later. I don’t think that these are lazy children, it’s not that they look for excuses not to participate in the sessions. Rather, I think they have totally internalized that they are not good doing PE, that they are not good at it, and I often observe how what I propose to them even leads them to feel anxiety. I have to try to get them to overcome this feeling of incompetence that they carry with them, and to this end, in each class I strive to make the climate in which the session takes place as affective as possible, to continually assess the efforts they make and to offer each person what they may need. I did not experience this as a student, and it has been difficult for me to accept myself halfway as I am”*.

(Teacher’s Diary. April 2010)

The above story corroborates that many of the subject’s objectors exist because they have learned to know and feel out of place within it. For this reason, designing activities in which all students have the possibility of enjoying successful situations can counteract the idea that PE is a vehicle at the service of excellence and, therefore, the heritage of an exclusive minority. In this sense, as an interim teacher, I am developing a teaching unit based on the ideas of the Pedagogical Treatment of the Body (see, for example, [42,43,44,45,46,47,48]) which aims to help schoolchildren learn to regulate their own efforts. As a result, I can see that it is the pupils who have the greatest difficulties in PE who see their feelings of motor efficiency reinforced to a greater extent if they experience these successful situations:

*“The best in the class in managing their effort have been an asthmatic pupil and an overweight pupil who show a high level of learned incompetence. Knowing that they were able to do the task better than the rest of their classmates was something that seemed impossible to them, and they were very happy. There are even days when they tell me that they want to dedicate the class to running”*.

(Teacher’s Diary. October 2009)

However, it is important to note that the condition of the inexperienced teacher implies the difficulty of knowing how to deal with certain didactic aspects [22,49,50], such as being able to attend to everything that happens in the classroom and knowing what is most relevant to consider in responding to the needs of each student. This is the case of the so-called invisible students [51,52], that is, those who try to go unnoticed during classes, a situation that I face in the Primary Education stage:

*“I have been observing the attitude of a six-year-old student for a long time. She tries to go unnoticed, as if she were an ‘invisible’ pupil. As it is difficult for me to keep an eye on everything that happens in the session, as there are almost twenty children who require my attention, on more than one occasion it has gone unnoticed. However, it is now one of my focal points, above all because it is a clear case of learned incompetence. Every time I propose a game or activity, however simple it may be, he tells me beforehand that he doesn’t know how to do it. Today, for example, we played “dodgeball” ; the first thing he said to me was that he didn’t know how to play, to which I replied that I had already played more times and knew perfectly well what it was about. But what he really meant was that he can’t dodge the balls that are thrown at him. Although we have changed the game so that the "touched" are not eliminated, but become pitchers, does not improve their involvement. As a pitcher, she is not considered good either, and she spends the game glued to the wall, just on the opposite side of where the ball is”*.

(Teacher’s Diary. October 2009)

In an attempt to overcome these difficulties that I encounter in the practice of the profession, I turn to the literature (see, for example, [53,54]) with the intention of learning more about the subject and being able to put new methodological options into practice. Thus, the idea of designing worksheets with the intention of recognizing those students with greater problems of self-esteem arises, under the personal conviction that education has to be a vehicle that favors the integral development of the whole student body. Furthermore, this resource makes me see that some children experience a phenomenon contrary to that of motor incompetence, that is, they believe themselves more capable of what they really are. Thus, it is not enough that, as an educator, I offer an appropriate response that allows students to experience situations of motor success. Rather, students must experience situations that they are not capable of overcoming. From these experiences, students will not only develop a tolerance for a lack of success, but also, fundamentally, capacities of perseverance and effort that favor their involvement by making them aware that learning and progress in their motor skills are taking place. It is here that it makes sense to leave room for the “teaching of error” [55] p. 87, in the classroom, provided that it does not have irreversible consequences for the student. That is, in situations that do not involve physical risk, students must be given the opportunity to learn by trial and error. The intention is to make the schoolchild aware of what they failed to do and to invite them to correct it, thus stimulating their curiosity and capacity to reflect and rectify. In my diary as a primary education teacher it is related as follows:

*“I continue to try to avoid, fundamentally with two first-cycle schoolchildren, the development of fears, fears and refusals towards physical practice because they believe they are incapable of doing what I am asking them to do. I design tasks in which they all go through situations of success, but where each one also experiences situations of failure. That is, no one is bad enough to not be able to overcome certain challenges, nor is anyone good enough not to keep learning and trying. With the second cycle students, the opposite is true, that there are two students who are not good at driving and yet they consider themselves excellent. I see that in situations where they are not able to overcome the challenge they are more aware of reality and see that they do not know how to do everything, which makes them try harder”*.

(Teacher’s Diary. November 2009)

The feelings of learned incompetence discussed above are not exclusive to primary school students. On the contrary, throughout my teaching career I have found that they are more common among secondary school students, because these students are at a crucial age for the development and strengthening of self-esteem and personal confidence. In addition, older individuals have low self-efficacy in relation to physical exercise and expect very little benefit from such activity, in contrast to younger people [39,56]. For this reason, as an educator I understand that the first responsibility is to try to create a pleasant and appropriate working environment. As Vaello explains [57] p. 9, “the secret of teaching is not so much to transmit knowledge as to transmit desire, especially to those who do not have it”. To achieve this, it is essential that schoolchildren can receive help and recognition from their peers, and from the teacher. This is hindered by the aforementioned public and easily observable nature of the subject [12], which is a burden on student participation in the teaching/learning process. This is expressed in my diary as a teacher as follows:

*“I have noticed for several days now that some students, always the same ones, as soon as they consider that the practice is difficult or that they might not be able to do it well, make excuses not to have to face it. Suddenly they tell me that yesterday they hurt their ankle, that their belly hurts, that they are dizzy, and this is happening at this point in the course when we are starting to work on strategies in collective games and sports. I know that what is happening to them is that they do not want their esteem to be damaged, they are thinking about what others will say or think”*.

(Teacher’s diary. February 2011)

The relationship that is established between the subject of this paper and the “sports perspective” [58] p. 160, partly explains the above position, because schoolchildren can believe that the result and victory are the main objectives of physical exercise. This leads them to behave in ways that places demands on the level of motor skills of the less skilled, who therefore tend to inhibit their practice. Among secondary school students this is a phenomenon that I observe with some frequency:

*“In today’s class, devoted to strategy in games, one student has refused to participate because she is not good at playing, and her classmates are continually scolding her for it. They know full well that I do not tolerate mockery of others, but it appears at times when they think I am not aware of it. So much so that this girl, although she is on the pitch, it is as if she were not there, and when, because of the changes I am making (including the fact that the ball circulates around the whole team before I can shoot at goal), it becomes essential that her teammates involve her, she immediately becomes nervous, exclaims that she does not know how to do it and gets rid of the ball immediately”*.

(Teacher’s Diary. February 2011)

To mitigate this type of situation, experience reaffirms my obligation to create a safe, ethical, democratic, and respectful learning climate, one that is adapted to the needs and possibilities of each schoolchild [43,48,59]. To do this, I propose situations of cooperative learning, understanding that with this type of work I could improve the autonomy of the students, socialization in the classroom, attitudes of solidarity, motivation towards the task, and positive development of the self-concept, while working on the objectives of the subject area [60,61,62,63,64]. The activities proposed are based on the fact that all the students must work together to achieve a final objective. Thus, those who are less tolerant of the limited capacity of their classmates are forced to focus part of their efforts on improving the capacity of others if they want to obtain positive results. 

It is also of interest that students are able to accept the possibilities of others and face, in a dialogical and respectful manner, the conflicts and difficulties that may arise in the development of activities that involve a high load of emotional tension (such as in the case of games and sports activities), because the tasks also have an opposition component: students work in two separate teams, so that the members of each team cooperate to oppose the other team. This manner of working offers results that, although not immediate and cause a feeling of unease at times, improves the feelings of motor competence and belonging among those who consider themselves less competent. An example of this evolution is found in the following accounts from my secondary school teacher’s diary:

*“In the different sets of the game in the student’s team with a low feeling of competence, discussions are frequent. Her teammates get angry at her clumsiness, and she gets angry at herself for her continuous mistakes during the game, which are bigger and more serious when the others reproach them, thus entering a vicious spiral. I find it hard to keep quiet and not intervene all the time, but I want them to design their strategies and fix the problems in an appropriate way”*.

(Teacher’s diary. February 2011)

*“I see that little by little they are making her more involved in the game, suggesting options to her and she is putting them into practice. I even hear some positive reinforcement from some of her classmates, of the "very well done" type. I’ve even seen her smile at others”*.

(Teacher’s diary. February 2011)

As I have described, the experiences that schoolchildren have in the classroom can affect their personal development positively or negatively, because these experiences are strongly imbued with psychological and social components (self-esteem, self-concept, acceptance by their peers, feelings of competence, among others). Thus, it is essential to awaken in teachers the sensitivity to place themselves in the position of the student who presents the most difficulties. Furthermore, this aspect must be worked on from the initial training stage.

### 4.2. Opening Up Perspectives on Learned Incompetence among Future PE Teachers

The imprint of the past, first as a student and later as a teacher in compulsory education, makes me aware of the importance of developing positive attitudes among students during teaching. This is an aspect I address in the initial training of university students during my period as an associate professor. 

It is clear, therefore, that what I seek for myself as an educator and what I try to be as a trainer of future teachers, is the same as what I seek for them: that they be reflective people, open to democratic perspectives, with values and an awareness of the importance of the profession they have chosen. As expressed by Contreras Domingo:

*“The teacher that I want to be with them I show them, so that they see it and I show them, so that they understand it, so that they embrace it, so that they apprehend it. But I also show them the master that I am (and this, inevitably, I do): they see me every day, with my questions, with my interests, with my provocations towards them, but also with my contradictions and difficulties), and I show them the students who are (sometimes happy, sometimes carefree, sometimes experts in the fictitious game of university teaching, in the tricks to pass without getting personally involved, without risking anything of themselves, sometimes sincere and passionate, sometimes disoriented with what I want from them and trying to translate it into conventional measures of school tasks, sometimes honest with themselves and with the commitment they want to assume). And I also show them the teacher I want them to be. And that in reality it only returns the question to them, asking them about the teacher they want to be so that they ask themselves seriously and thoroughly”*.

[65], p. 16

Thus, I understand that future teachers, before worrying about what they will teach, have to ask themselves who they are as people and identify the ethical, political, and cultural dimensions, among others, that will govern their teaching [66]. I am mainly interested in their ability to respond to situations in which schoolchildren may germinate negative attitudes towards themselves. Hence, I am opening a debate in which we can put forward different points of view, ideas, and conceptions that will help find resolutions to some of these issues; as my associate professor’s diary states:

*“I have proposed the reading of two articles that deal with the problem of the lack of interest of the students towards the subject of PE and the feelings of learned motor incompetence. Next week we will devote part of the practical class to discussing this, and I ask you to draw up a sketch in which the main ideas of these articles are reflected. I am trying to get them to develop strategies that they will have to put into practice in the near future and, above all, to shape the type of teacher they want to become: whether one who is committed to the students, or one who has chosen the profession because he or she considers it simple and well-paid”*.

(Professor’s Diary. April 2011)

My responsibility, at this stage as a university teacher, is therefore aimed at trying to form a collective of future teachers who will contribute to the construction of a more just and equitable society by proposing debates and sharing that will serve to open up perspectives and points of view and, consequently, help future teachers to be aware of the responsibilities inherent in the profession. This is how we derived the *“Ten commandments for the development of motor skills and to increase students’ self-esteem”*, a title collectively suggested by the class group and which reflects the main essence of the subject. The following measures were considered in this decalogue which, as future educators, should be taken into account to avoid or at least minimize the consequences of a low feeling of motor competence among their future students: (1) The PE teacher must carefully observe the reactions and behaviors of their students and of the students themselves, because gestures and attitudes offer a large amount of information to address dilemmas that the students may face in a timely manner. (2) The responsibility for fostering feelings of competence is not exclusive to the PE teacher. The teacher must be supported in their work by their families and the wider teaching staff at the school, who must actively collaborate with them to compare data and opinions. (3) To take care of the manner in which the teacher addresses the students, because, consciously or unconsciously, the teacher can reinforce ideas and feelings among the students that do not benefit the positive development of the students’ self-esteem. Positive reinforcement must be present in all sessions and, in particular, it must be directed at those who face greater difficulties, to highlight their qualities in front of their peers. (4) To favor a good climate in the classroom, tackling the comments, mocking, and attitudes that can condition the participation and positive use of teaching sessions. (5) To select varied and attractive activities in which the interests of the students are taken into account, without stereotyping or a main objective of victory. Schoolchildren must bear in mind that the primary objective of PE must be to learn, not to win. (6) To provide opportunities for all schoolchildren to improve their level of motor skills in PE, ensuring that the most skilful will help those who have the most difficulties in practice, and adapting the rules of games and sports where necessary. (7) To promote productive and educational diversity in the classroom because this allows learning from colleagues. (8) To delegate part of the teacher’s responsibility to those students who have less self-esteem and self-confidence as a means of showing confidence in their abilities. (9) To provide the necessary time to carry out the proposed activities: less can be more. (10) To carry out a continuous assessment of students with greater difficulties in practice in which the individual progress achieved prevails over other criteria.

This type of work leads me to consider that what is important at this stage is not so much that students learn to teach but that they consider teaching as something more than the mere transmission of knowledge. That is, that they understand that “education is a slow journey [...] in which people go through a process that helps them grow intellectually and emotionally” [67], p. 82. This concern is reflected in the following account from my diary as a university professor:

*“In class we work on issues similar to those they will have to face in their day-to-day classroom life, aspects related to discipline, motivation, low involvement of school children and the feelings of helplessness that some of them show. The intention is to make them aware of the responsibilities inherent in the profession they have chosen and to develop a critical, fair and equitable sense. It is not in vain that their future work involves training the citizens of tomorrow, people for whom it is essential to strive to make them capable, full and happy with themselves”*.

(Professor’s Diary. April 2011)

This idea is part of the intention to train good professionals. As Bain explains about the best teachers:

*“They are the ones who talk about helping learners to strive with ideas and information to build their own knowledge. […]. While others may be satisfied if students do well in exams, the best teachers assume that learning makes little sense if it is not able to produce a lasting and important influence on the way people think, act and feel”*.

[68], p. 28

## 5. Conclusions

In the previous discussion, I described the feelings and concerns of a new PE teacher relating to the usual feelings of motor incompetence among students. Thus, I described how my experiences, first as a student, and then as a teacher in initial training and at different educational levels, opened in me a deep sensitivity and conviction to eradicate this type of attitude among schoolchildren. Two of the basic pillars in a correct teaching performance to work towards achieving this are (i) being receptive to the circumstances experienced by students and (ii) trying to adapt learning situations to provide all students with the possibility of experiencing successful situations. Ensuring that students experience successful situations, and reaffirm their confidence and personal self-esteem, leads not only to a greater disposition towards learning, but also to academic improvement within the subject [69,70,71].

In this regard, Ruiz Pérez states that:

*“To propose a more equitable learning in which all students can have opportunities to receive rewards and taste success, success that must be redefined in terms of personal improvement and not of improvement of others”*.

[3], p. 130

The reflections and experiences of my third year in the teaching profession, within the primary education stage, provided the necessary lucidity to understand that experiencing motor success depends to a great extent on the opportunities for practice that are offered to schoolchildren and the manner in which these are presented to them. This ratifies the idea that success is often only possible in circumstances in which it is essential to beat others (for example, games in which participants must be faster than others to avoid being caught, or sports activities in which a successful dribble or an effective throw is necessary to beat the opponent). In these situations, it makes sense to propose learning in which students know how to respond positively to opposition and high emotional involvement, without this posing a threat to their self-esteem.

In these cases, practical knowledge is centered on making it clear to students that the achievement of a proposed task must never be experienced as an affront to another person, nor as an affront to one’s own ego. Achievement must be considered as an event that is inherent to practice, and, if not possible today, provides an idea of what, with effort and perseverance, will be possible tomorrow. To achieve this, as in previous courses, strategies based on positive reinforcement and high expectations of the students are needed. 

It is important to note my predisposition, despite being an inexperienced educator, to reinforce the positive expectations of all students. Nonetheless my teaching practice reaffirms that I tend to carry out closer and more personal monitoring of the situations of those students who present lower self-esteem and, consequently, a greater feeling of motor incompetence. I believe, therefore, that there is the possibility of favoring these “weak subjects”, thus neglecting those who are more motor competent. For example, in sessions during which I notice that certain schoolchildren present more difficulties, I make a greater number of adaptations, so that those less able can cope successfully. However, providing a greater number of situations in which less able students can feel comfortable and competent diminishes the challenge for those who are more gifted. This creates a risk, as stated by Rogers [72], of stifling the intrinsic motivation of more able students, and diminishing their involvement and taste for the subject. 

As a result of this experience, I consider it valuable for future physical educators to ask themselves questions such as: “do my past experiences as a schoolboy condition my approach to teaching so much that these experiences result in burdening the most capable students?”, and “is it not my responsibility that each student offers the best of themselves?”.

This feeling of uncertainty, and of not being able to balance the treatment and responses needed by each schoolchild according to their possibilities and limitations, leads me to understand that it is my responsibility to make PE a satisfactory experience for all students. This is a current focus of my teaching practice because I understand that achieving competency, both in education and in the area of motor skills, involves giving all students a certain amount of autonomy.

To do this, it is essential to design tasks in which schoolchildren have to use their perceptual-motor resources, offering them varied and abundant opportunities for practice, while at the same time ensuring that the practice contexts create dissonance [3], i.e., the practice opportunities are one step ahead of what the students are capable of doing. 

It is also important to remember that, to improve teaching practice, educators need to be clear that:

*“Becoming competent in PE must be a joyful, creative and productive undertaking that must be studied in the places and contexts where it is carried out, and in which teachers and researchers must maintain a close relationship, and in which teachers must be researchers of their own reality”*.

[3], p. 134

Finally, when working on some of the content of the subject (e.g., the development of motor skills or activities with a sporting character), as trainers of future educators, questions come to mind that concern us and can open a dialogue between teachers: Do past experiences as a schoolchild condition our approach to teaching so much that they result in limiting the most capable students? What role can autobiographical narratives play in better understanding our past as students and, consequently, in guiding our future pedagogical practice?
